# Construction Site Safety Management: A Computer Vision and Deep Learning Approach

**DOI:** 10.3390/s23020944

**Published:** 2023-01-13

**Authors:** Jaekyu Lee, Sangyub Lee

**Affiliations:** Energy IT Convergence Research Center, Korea Electronics Technology Institute, Seongnam-si 13509, Republic of Korea

**Keywords:** worker safety management, virtual datasets, synthetic datasets, image processing, transfer learning, virtual validation environment

## Abstract

In this study, we used image recognition technology to explore different ways to improve the safety of construction workers. Three object recognition scenarios were designed for safety at a construction site, and a corresponding object recognition model was developed for each scenario. The first object recognition model checks whether there are construction workers at the site. The second object recognition model assesses the risk of falling (falling off a structure or falling down) when working at an elevated position. The third object recognition model determines whether the workers are appropriately wearing safety helmets and vests. These three models were newly created using the image data collected from the construction sites and synthetic image data collected from the virtual environment based on transfer learning. In particular, we verified an artificial intelligence model based on a virtual environment in this study. Thus, simulating and performing tests on worker falls and fall injuries, which are difficult to re-enact by humans, are efficient algorithm verification methods. The verification and synthesis data acquisition method based on a virtual environment is one of the main contributions of this study. This paper describes the overall application development approach, including the structure and method used to collect the construction site image data, structure of the training image dataset, image dataset augmentation method, and the artificial intelligence backbone model applied for transfer learning.

## 1. Introduction

There are a variety of risk factors in construction sites, and injuries frequently occur from workers not wearing protective equipment or a lack of proper safety training [1]. Therefore, various studies are being actively conducted to reduce risks and accidents occurring at work sites using IoT devices and technologies such as computer vision, big data, and artificial intelligence [2,3,4,5,6]. Additionally, research on preventing worker collision accidents using ultra-wideband (UWB) communication-based worker location data is in progress. However, worker safety management systems using Internet of things (IoT) devices are inconvenient, as each must be worn by workers. Camera-based safety management systems do not require the workers to wear IoT devices, allowing for efficient prevention of worker safety accidents. In this regard, more research is necessary for developing image-based safety management systems using cameras. Similarly, such research is being conducted based on images collected from CCTV cameras installed at construction sites to prevent accidents involving construction workers [7,8,9]. In particular, object recognition technology is widely used for the safety management and security of workers at a construction site. Object recognition technology, which recognizes various objects in image frames that are input through a video, has been researched for many years in the field of computer vision, and object recognition is a commonly used technology [10,11]. This study was conducted to reduce safety accidents for workers at construction sites using deep learning-based object recognition technology. In addition, three image recognition models were developed to prevent safety accidents involving construction workers. Moreover, the developed image recognition models were used to develop a CCTV-based safety management application. Data consisting of many images of workers were collected from construction sites and used to train the object recognition models. The object recognition models optimized for construction sites were created using the collected image data and transfer learning technology. The first model recognizes worker objects, and determines whether construction workers are present at the construction sites. This object recognition model is used to recognize workers wandering around or trespassing in a work site outside the working hours. The second object recognition model perceives workers who are falling off a structure or falling down. In particular, this model determines whether a worker has fallen from a height by estimating the pose of the worker. The third object recognition model determines whether workers are wearing safety equipment. It mainly determines whether workers are wearing hard hats and generates a warning notification when workers not wearing hard hats are recognized. This paper is structured as follows. Section 2 presents a literature review of studies on image-based technology for construction site safety management. Section 3 describes the object recognition models and the overall system that we developed for construction site safety management. Section 4 presents the implementation of the application using the trained models and the results of testing the application. Finally, Section 5 provides some concluding remarks and areas of future research.

## 2. Related Work

Currently, studies for improving the safety of the construction sites based on computer vision technology are being widely conducted. In [8], an image recognition system was proposed to improve the safety and productivity of tower cranes operating at construction sites. In that study, a camera-based image recognition embedded system was developed that could be mounted on a tower crane. Although the latest deep learning-based vision technology was not utilized, it was indicated that applying the developed system to tower cranes can save a considerable amount of time by preventing delays occurring from the work environment, lighting conditions, and blocked views. A study was also conducted on combining semantic inference with computer vision technology for construction site safety management [12]. A framework was presented that combines computer vision and ontology technologies for managing the safety of construction workers. Computer vision technology is used to analyze visual information in images from construction sites. By comparing the visual information extracted from construction site images with the predefined Semantic Web Rule Language rules, construction site risks are inferred. That study demonstrates that it is possible to identify and prevent risks on construction sites by recognizing the images of the construction site workers based on the proposed framework and inferring hazardous situations through a semantic algorithm [12]. In addition, a study was conducted regarding the development of a real-time computer vision system for recognizing the helmet and safety gear of the worker in an actual construction site and estimating the worker’s pose [13]. The study proposed a computer vision system that can detect construction site workers and their personal protective equipment in real time. In that study, 95 video datasets were constructed based on the data collected from an actual construction site, and an object recognition model was created using Python in a TensorFlow environment [13]. Moreover, research was conducted on the design of a framework for construction site safety management using deep learning and computer vision technologies. In that research, unsafe actions by workers and the working conditions at the construction site were identified using the proposed framework [14]. A study on the development of an image-based automated monitoring system for efficiently managing whether workers are wearing safety equipment at the work site was also conducted [15]. In that study, a deep learning object recognition model was used to determine whether workers were properly wearing their personal safety equipment while dismantling the Fukushima nuclear power plant. Furthermore, a methodology for recognizing worker safety equipment was proposed, and a corresponding model was developed based on the collected data and the proposed method conducted in the study [15]. In addition, a study was conducted to determine whether the rules for wearing safety glasses are adhered to in a learning factory, where safety risks occur. Such determination was made by combining the vision technology of Microsoft Azure and an artificial intelligence (AI) service. The model provided by the Azure platform was used as the artificial neural network [16]. Moreover, a framework was designed that can recognize, in real time, whether construction workers are complying with the safety rules by recognizing the personal protective equipment based on images [17]. A new neural network model for safety management was also developed by applying transfer learning to the YOLOv3 model. That research emphasized that the wearing of personal protective equipment for workers is even more important because safety risks occur in a construction work environment [17]. Moreover, a study conducted a critical review on the safety monitoring of construction site workers based on computer vision technology [18]. That study indicated that computer vision technology is considered an effective solution for achieving safety at a construction site. However, it also examined the computer vision technology utilized at construction sites and presented the limitations from technical and practical aspects [18]. As such, computer vision technology has been researched within a wide range for the prevention of safety accidents at construction sites, and is being applied either directly or indirectly to actual construction sites. Object recognition technology based on computer vision and deep learning is being actively investigated in various fields, including construction site safety. The automotive field is the typical research field in which object recognition models have been widely investigated. Various studies have been conducted to further development object recognition models for the autonomous driving of vehicles based on deep learning technology. In one study, a neural network model was used to generate a steering command for a vehicle based on the lane detection data of an object recognition model. The model does not merely recognize lane objects but uses a neural network model to generate steering commands based on lane data recognized by an object recognition model [19]. Furthermore, although this is not a study related to safety improvement at construction sites using computer vision and deep learning technology, which is the research direction of the current study, improvements to the performance of the object recognition model were attempted. A study was conducted to solve the inefficiency problem of a recurrent attention convolutional neural network (RA-CNN), which selects a single feature region and recursively learns the features of the region. In that study, a novel fine-grained visual recognition model was established, i.e., a multifeature RA-CNN, which associates multiple feature regions to overcome the inefficiency of RA-CNN and improve the classification accuracy. Additionally, a feature scale-dependent algorithm was developed to improve the classification accuracy, and the performance of the developed algorithm was verified using the three most popular benchmarks: CUB-200-2011, Cars196, and Aircrafts100 [20]. In addition, object recognition model development using machine learning technology with multiple linear regression (MLR) has been investigated. In particular, a machine learning model was developed to recognize objects and human faces using three machine learning algorithms: linear discriminant analysis, fuzzy inference system, and fuzzy c-mean clustering. Fuzzy c-means clustering was combined with an MLR function to reduce four-dimensional variables to two-dimensional variables, and MLR was applied to logistic regression to minimize the outlier. The disadvantages of each machine learning model were mitigated by combining machine learning models with MLR, which additionally afforded a lower processing time compared with using deep learning object recognition models [21]. In this study, we focus on developing an object recognition model for construction site safety management using a synthetic dataset based on transfer learning and improving the safety of construction site workers by utilizing the developed object recognition model instead of focusing on improving the performance of a single algorithm. Moreover, we prioritize the development structure and procedure of the object recognition model to improve the safety of construction workers, safety management scenarios, synthetic dataset acquisition, object recognition model development based on transfer learning, and model verification using a virtual environment.

The most significant differences between this study and previous studies are in the areas of data acquisition, model learning, and model validation. In terms of data acquisition, object recognition models reported in previous studies were primarily created using only image data acquired directly from construction sites. To create an image object recognition model of a construction site or to improve the performance of an object recognition model, a significant amount of image data must be obtained, and the method of directly acquiring image data at the construction site is inefficient. In this study, we directly create a synthetic dataset for learning an object recognition model based on a virtual environment. This method solves many problems associated with data acquisition. Next, we develop an object recognition model for construction site safety management based on transfer learning. Because transfer learning technology uses a prelearned model, the learning time can be reduced and an efficient object recognition model can be learned using a small amount of image data. An efficient object recognition model can be developed rapidly using a transfer learning algorithm when developing an object recognition model for a new domain in which acquired and accumulated data are insufficient. Finally, it is an environment for model verification. Testing developed object recognition models is difficult, particularly object recognition models pertaining to worker fall detection. In this study, we test an object recognition model for worker fall detection based on a virtual environment and experimentally verify the possibility of creating a virtual verification environment. This verification method is regarded as extremely efficient and can be realistically tested before it is applied to an actual construction site.

## 3. Development of Object Recognition Models for Construction Site Safety Management

In this section, we comprehensively describe the overall development approach of the object recognition models for construction site safety management, including the development environment, the procedure for collecting the image data for training, the image preprocessing technique, the structure of the image datasets, the backbone model, and the development of the construction worker safety management model.

### 3.1. Development Structure and Procedure

**Figure 1:**
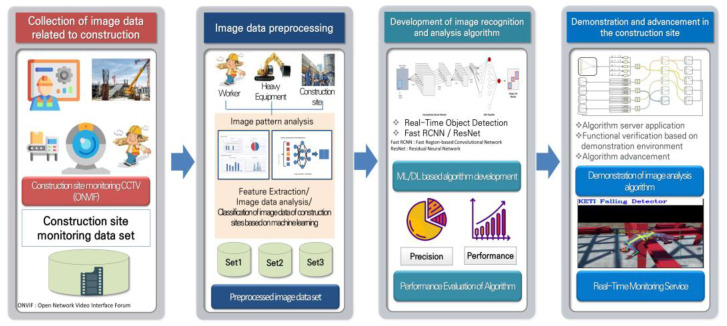
Development of CCTV-based image recognition and analysis technology for worker safety.

Figure 1 shows the structure and process of the image analysis model developed for an intelligent image analysis of a construction site, and the development structure and process are briefly described as follows. First, construction image data are collected through construction site CCTV, Internet crawling, and public data. The collected image data are then converted into high-quality data through preprocessing and stored on the server. Next, the stored dataset is structured into a dataset required by the learning model, and transfer learning is conducted using a previously trained model based on the structured dataset. Finally, the generated model is tested and verified, and the construction site safety management system was developed based on the verified model. Transfer learning is an artificial intelligence technology that creates a deep learning model by training with a new dataset based on the weights of a model trained on a very large dataset such as ImageNet [22]. Building image data to create a new model and training the model starting with the initial values require significant time and resources. However, an artificial intelligence model can be created with a small dataset when creating a new model based on transfer learning because the object recognition model already has the trained weights (*.Weight) [23,24]. Even Google’s GoogleNet [25] and Microsoft’s ResNet [26] have not been trained with all data. It would therefore be efficient to derive a new model by applying only the dataset that is to be classified to the previously trained model, as used in transfer learning. To conduct the transfer learning, a basic model, called a backbone model, is required. Various models, such as VGGNet [27], YOLOv3 [28], ResNet, and ImageNet, are used as backbone models. Because a VGGNet or ResNet model has been trained with the feature vectors of the convolutional layers, only a single layer needs to be trained again based on the customized data. Models generated in this way have an advantage in that they achieve a better performance and significantly faster training speed than models that have been trained from the initial state. In this study, the ResNet model was used as the backbone model to create object recognition models for preventing safety accidents involving workers at construction sites.

### 3.2. Development Environment and System Configuration

The development environment (hardware/software) of the transfer learning-based object recognition model for preventing safety accidents involving construction site workers is summarized in Table 1.

The hardware platform used to train and operate the artificial intelligence model is as follows. As shown in Table 1, an AMD Ryzen 9 3950x CPU, 64 GB of RAM, a high-performance GeForce RTX3080 GPU, and 1 Terabyte of NVMe storage were used. Because three models will be operated on a single platform, in addition to training the artificial intelligence models, high-end CPUs and GPUs were used. Furthermore, the software development environment is briefly described as follows. The operating system is an Ubuntu 18.04 environment, and the virtual environment for developing the artificial intelligence models was developed using Python 3 (Python 3.8.0) and TensorFlow (1.15.2) based on Anaconda 4.9.2. Because OpenCV is a programming library for real-time computer vision processing, it was used to process the images more efficiently [29]. Moreover, a virtual environment was built in Unity (2019.4.2.f1) to augment the image data. After creating 3D virtual image data such as helmets in the virtual environment, the data were converted into two-dimensional data, and the image data were accumulated.

### 3.3. Image Data Collection

**Figure 2:**
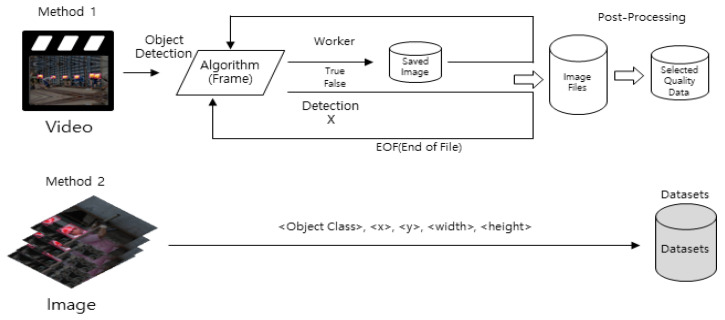
Procedure for collecting construction site data.

A large number of training data need to be collected to create an object recognition model for a construction site. Figure 2 shows the procedure for collecting the image data required for training.

In this study, construction site image data were collected using videos, pictures, and web crawling, and the procedure for image data collection is as follows. First, the collected video file was segmented into each frame, from the first frame to the last frame of the video. Among the segmented frames, only the frames in which a worker object exists were used for training, and were saved in a picture format. Images (frames) that had been classified from the video were labeled as part of the MS COCO dataset [30] structure for ResNet training. Next, only those pictures that contained workers or helmets, which are needed for training, were classified from the collected image files and labeled as the MS COCO dataset structure. In addition, over 15,000 pictures of image data were collected through web crawling, and these images were also labeled according to the MS COCO dataset structure and then used for training. The image data collected through public data consists of a total of 32,150 photographs.

**Figure 3:**
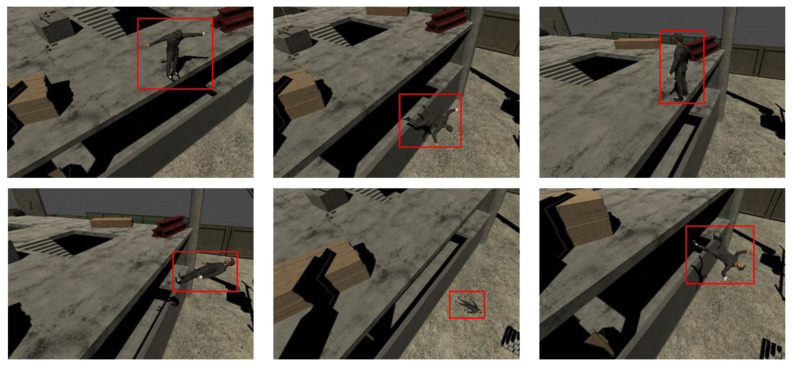
Image data collection based on virtual environment.

In this study, a virtual construction environment was constructed to collect a more abundant image dataset, and a variety of image data (construction workers, hardhat, etc.) were collected from the virtual construction site. The virtual environment was constructed using Unity tools, and the physics engine provided by the Unity development environment was used to simulate the fall of workers and movements at the state of fall injuries. Figure 3 presents some of the image data collected based on the virtual environment, and the red bounding box shows the collected construction worker data. In this method, collecting image data based on the virtual environment allows an efficient construction of a large image dataset, and the developed object recognition algorithm can be tested numerously based on the virtual environment to derive the optimal algorithm.

### 3.4. Preprocessing Module

The preprocessing module used for creating the models for construction site safety management is as follows. First, image binarization is performed as shown in Table 2. Image binarization is a technique that converts videos or images into black and white based on the given threshold value. Image binarization is used to distinguish the objects from the background before applying the image processing algorithm [31]. The threshold value that minimizes the classification error owing to a binarization is called the optimal threshold. Algorithms such as the locally adaptive thresholding algorithm, hysteresis algorithm, and binarization considering timescale have also been developed.

A grayscale preprocessing algorithm was used to convert the color of the videos or images into gray. The algorithm is used to remove color values when colors are not needed in image processing. The grayscale values were divided into a total of 256 levels, from 0 to 255, based on the light intensity. Black color is expressed as 0, whereas white is expressed as 255, and gray, which is the middle level, is expressed as 128. When an image is converted into grayscale, the data used for expressing color can be reduced. As a result, the efficiency of the classification increases, and overfitting can be reduced when training with images [32]. Next, an image pyramid module was developed to enlarge or reduce the images. The image pyramid algorithm changes the size of the image so that it can be sampled efficiently at the desired level [33]. In other words, this module can be used to enlarge or reduce the size of an image to the desired level. In addition, an image rotation module was designed to rotate the image data collected from the construction sites at various angles (e.g., 90°, 45°, and −45°). This module is used to correct the images used in training and rotate the images at various angles. Other preprocessing modules include a histogram and the image segmentation module. A histogram is a frequency distribution table, and is a graph that divides the data distribution into several segments and visually expresses the data belonging to each segment. The features of the image can be more easily identified by expressing the pixel values of the image as a histogram [34]. Image segmentation refers to segmenting a digital image into several regions, and has been used to classify meaningful objects in the construction site images [35]. In addition, the image segmentation module finds important parts in the construction site images and is used to collect image objects needed for transfer learning. Table 3 shows the preprocessing results of the image segmentation module.

### 3.5. Image Dataset Structure

In this study, we created object recognition models by applying transfer learning based on the ResNet backbone model. To conduct this transfer learning, the structure of the training data needs to be modified to match the MS COCO dataset structure. This section describes the training data structure for each model. DarkNet is an open-source object recognition neural network framework written in C language. The DarkNet YOLO dataset refers to the image dataset required for training the YOLO neural network. The DarkNet YOLO dataset is classified into training data and test image data. The training dataset comprises the image files to be trained, the labels of the objects, and text files (*.txt) indicating the region of the image to be trained. These text files contain information including the object class, the location of the object (x, y), and the size of the object (width, height). The lines are added according to the number of objects used to express the information.

Keras is an open-source neural network library written in Python, and was developed in the Open-ended Neuro-Electronic Intelligent Robot Operating System (ONEIROS) project. This library is used for training and testing Keras-based neural networks. The Keras image dataset is divided into training image data and test image data folders. Each dataset comprises the image files, the text files (_class.txt) indicating the object labels, and the text files (_annotations.txt) indicating the region of the images to be trained [36]. Unlike the DarkNet dataset, which saves the region to be trained in each image as a separate text file, the Keras image dataset defines the regions of the images to be trained in a single text file. This text file defines the image name, the starting coordinates (top-left corner of the bounding box), and ending coordinates (the bottom-right corner of the bounding box) of the object to be trained, as well as the object class.

**Figure 4:**
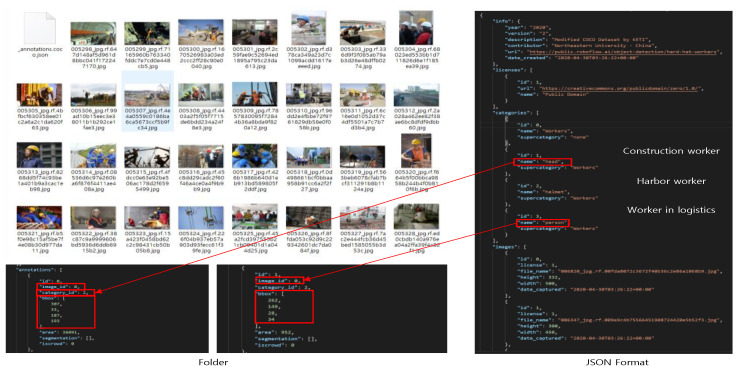
Structure of the Microsoft COCO dataset.

Next, the Microsoft Common Objects in Context (COCO) dataset is a dataset used for object detection, object segmentation, and key point detection. In addition, universities and companies around the world use the COCO dataset in diverse ways. In addition, the COCO dataset folder contains many different image files, as shown in Figure 4, and information about each image object datum is defined in a single JavaScript Object Notation (JSON) file format. In addition, the Pascal Video Object Class (VOC) dataset is an image dataset used in object class recognition technology competitions to evaluate the performance of object recognition models. Basically, the Pascal VOC image dataset consists of an image dataset for training and an image dataset for testing. Each dataset folder contains image files and Extensible Markup Language (XML) files describing the information about the objects in each image. The XML files are defined by the location of the image folder, the name of the image file, the path to the image file, the object class, and the X-coordinates (xMin, xMax) and Y-coordinate (yMin, yMax) of the object [37].

Lastly, TensorFlow is an open-source library developed by Google for both deep learning and machine learning. This library is widely used in artificial neural networks, including image object recognition. The TensorFlow-based image training dataset comprises image files and an Excel file. The Excel file contains the name of each image file, the class of the objects corresponding to the image file, the class size (width, height), and the location (x min, y min, x max, y max) data.

### 3.6. Backbone Model

ResNet is the backbone model used for creating the safety management object recognition model based on images to reduce safety accidents involving construction workers. ResNet is a convolutional neural network (CNN) model developed by Microsoft for image classification. This model has residual blocks, and thus there are shortcuts to add weights to the input and output values. As a result, the performance of the ResNet model tends to increase in proportion to the depth of its neural network. In other words, the existing neural networks aim to obtain function H(x), which maps the input value x to the target value y. However, the objective of the ResNet model is to minimize F(x) + x. This model is called ResNet because it is an algorithm that minimizes the residuals. In addition, ResNet is basically a structure in which shortcuts are added after increasing the depth of the network by adding convolutional layers to the VGG-19 neural network.

### 3.7. Model Creation

**Figure 5:**
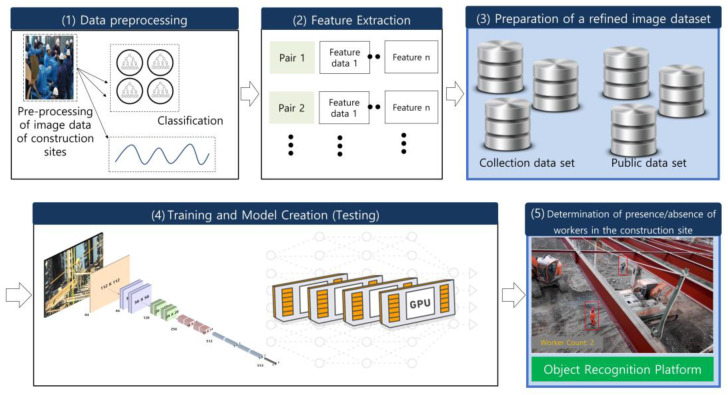
Development of the object recognition model for recognizing workers at a construction site (Model 1).

The object recognition model was created based on transfer learning to distinguish workers at a construction site using the collected image data. ResNet was used as the backbone model in transfer learning, and the learning procedure is as shown in Figure 5. First, data consisting of images of workers at construction sites were collected. The collected images were then refined by applying the preprocessing module to them. Next, data needed for training were extracted based on the features of the image data (e.g., worker, face, and image distribution). The extracted data were processed to fit the MS COCO data format. Finally, transfer learning was conducted based on the refined images and training data used to create the model. The ResNet backbone model used in transfer learning consists of 152 layers. In general, the performance of the neural network does not increase simply because the neural network is deep. However, because there are shortcuts in the ResNet neural network, the network has been designed to improve the performance in proportion to the network depth. ResNet is basically a structure in which shortcuts were added after increasing the depth of the network by adding convolutional layers to the VGG-19 neural network.

**Figure 6:**
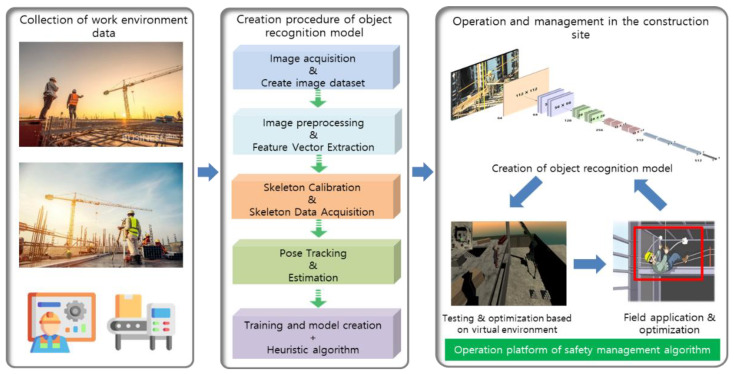
Development of the model for preventing the fall (falling off or falling down) of construction workers (Model 2).

To determine whether a worker operating at a height has fallen down or fallen off a structure, the fall of the worker is detected based on the pose estimation algorithm. This model classifies the state of whether a worker has fallen into “normal,” “warning,” and “danger” categories, and Figure 6 shows the procedure used for developing this model.

The pose estimation dataset, as well as the image dataset, were collected to create a model for preventing falls involving construction workers. For the pose estimation dataset, data were collected based on the MPII Human Pose dataset [38] and DensePose-COCO [39] datasets, which are commonly used in the field of pose estimation and prediction. The MPII Human Pose dataset consists of 25,000 images containing 40,000 people. This dataset also provides annotations and labels for 410 human activities, which have been analyzed and classified based on YouTube video data. The structure of the joints in the human body was mainly analyzed in the collected dataset. In particular, training was conducted based on the tilt of the knee, the position of the head, and the position and direction of the torso to create a model for detecting a fall from a structure or falling down of a construction worker. Furthermore, the model was designed and implemented using a “top-down” method, which first recognizes a person in the image and then estimates the person’s pose inside the bounding box, to improve the fall detection performance. This design method can improve the object recognition accuracy compared to a “bottom-up” method, which estimates the key points of a person in the image and analyzes the correlation between the key points to estimate the person’s pose. ResNet was also used as the backbone model of the encoder and decoder in the model used to detect fallen workers. Moreover, the Hourglass model was also used to capture various pose information at different scales.

**Figure 7:**
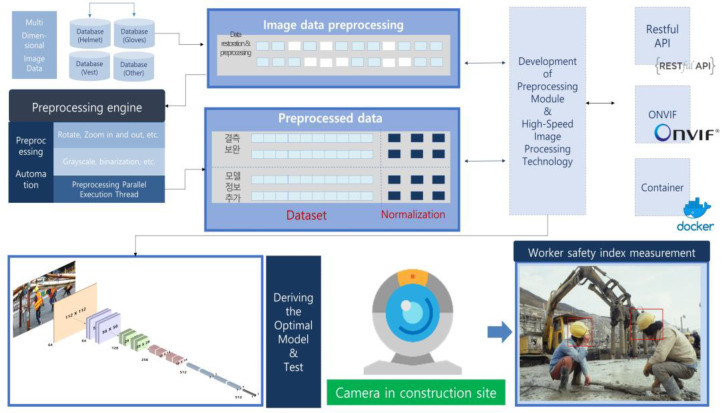
Development of the construction worker safety index measurement model (Model 3).

One of the eight major reasons for accidents that occur at construction sites is workers not using the safety equipment that has been provided to them. The safety index measurement model recognizes the worker’s safety equipment (e.g., helmet and safety vest) and determines whether the worker is wearing proper safety equipment. Figure 7 shows the structure and procedure for the development of the worker safety index measurement model. As shown in Figure 7, the collected image dataset is converted into images optimized for training and creating the model through preprocessing. In addition, the model was designed to enhance low-resolution images. Furthermore, the Unity virtual environment was created to increase the training data, and the amount of training data was drastically increased by creating virtual helmet images based on the virtual environment. The backbone models used in designing the construction worker safety index measurement system are ResNet18, ResNet34, and ResNet101, among others. After conducting various tests, a new safety index measurement model was developed based on the ResNet 101 backbone model. In other words, the weights were used as they are for the middle layers of the backbone model, and only the last 512 fully connected layers were trained anew to reproduce the image analysis model.

### 3.8. Internal Structure of Object Recognition Model and Data Flow for Each Model

**Figure 8:**
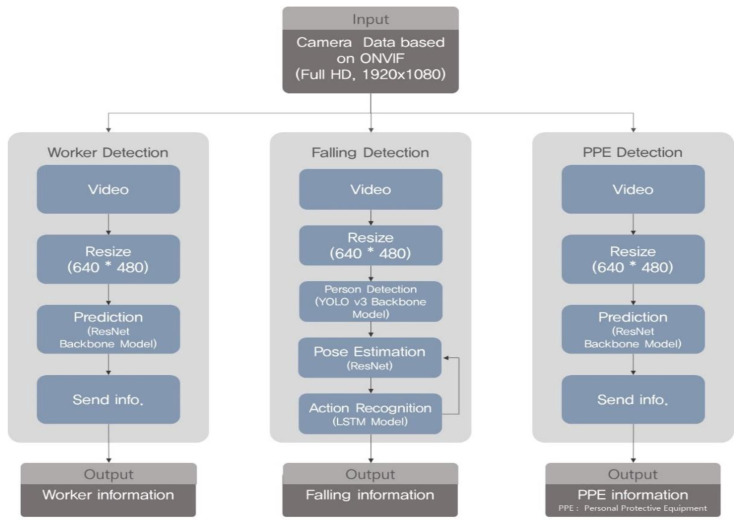
The detailed structure of object recognition algorithm.

**Figure 9:**
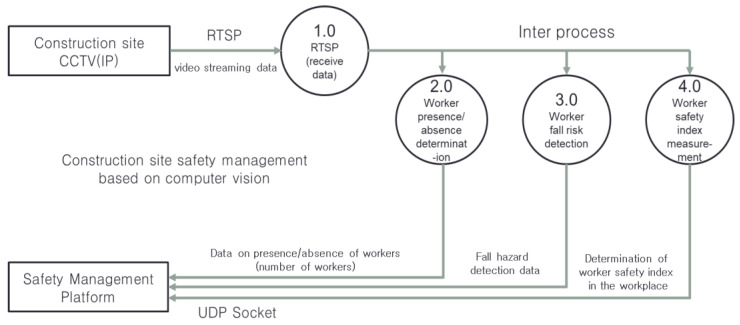
Data flow of construction site safety management algorithm.

Figure 8 shows the specific structures of each object recognition model. From the left of the figure are the specific structures and execution procedures of (1) the worker detection algorithm, (2) the worker fall detection algorithm, and (3) the algorithm for the determination of the wearing of personal protective equipment. The execution structure of the worker object recognition model and the personal protective equipment (PPE) object recognition model for determining whether a safety cap is worn are very similar. The worker object recognition model and the PPE object recognition model recognize worker and safety hat objects from the Full HD video received through the Open Network Video Interface Forum (ONVIF) protocol. The image data input to each algorithm is adjusted to a resolution of 640 × 480 suitable for the input size for the object recognition model. The object recognition model detects from each frame the worker and safety hat objects in the image adjusted to 640 × 480 resolution. The detected objects were designed to be output to the screen through the user interface module. In addition, the execution procedure for the worker falls and fall injuries detection model is as follows. Through the one-class YOLO v3, which only recognizes human objects, only the human objects within the frames are recognized, and the recognized data are delivered to the posture estimation model. The ResNet-based posture estimation model extracts the skeleton-pose data from human objects, and the workers’ movements are predicted every 30 frames in the LSTM action recognition model based on the skeleton-pose data. The workers’ falls are detected in the LSTM model based on the predicted movements, and the workers’ falls are determined. Figure 9 shows the simplified data flow of the algorithm pertaining to the safety management of construction sites. First, the safety management algorithm receives video data from the construction site CCTV based on the ONVIF protocol, and when an (accident) event occurs, the message is transmitted to the safety management platform via the TCP/IP (UDP) protocol. In addition, the worker detection algorithm calculates the number of workers at the construction site and continuously transmits data to the safety management platform, as shown in Figure 9 (2.0). The worker fall detection algorithm (3.0) detects the fall of a worker at a construction site. At this time, the walking, standing, and sitting motions of the worker are considered normal, and a fully fallen state is assumed to be a risk (danger). The case where the worker is not in a normal or dangerous state and the case where the worker’s posture changes rapidly are judged as warning states. The danger signal detected by the worker fall detection algorithm is transmitted to the safety management platform via the UDP socket. The worker safety index measurement algorithm determines whether workers at the construction site are appropriately wearing safety equipment, such as hard hats and safety vests. When workers who do not wear protective equipment properly are present at a construction site, a danger signal is transmitted to the safety management platform.

The algorithm proposed herein allows one to verify whether workers enter and exit the construction site outside of working hours, thus promoting worker safety via unauthorized worker access control. Next, an initial response is realizable when a fall accident occurs to a worker, i.e., the worker can be transported immediately to a hospital by the safety manager at the construction site. In addition, worker safety can be improved by preventing workers from entering construction sites without safety equipment. However, the proposed algorithm does not completely and automatically improve the safety of the construction site but can efficiently improve the safety of workers through the safety manager.

## 4. Implementation and Simulation

This section describes the implementation of the application for construction site safety management using the developed object recognition model. The interface design for video reception, verification of the created model, and the simulation and test results of the implemented system are described.

### 4.1. Data Flow and User Interface Design

**Figure 10:**
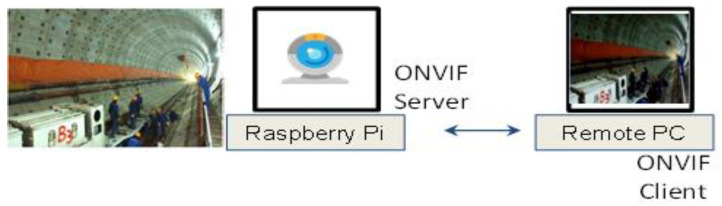
Development of the video data communication module based on the ONVIF standard.

The object recognition model for construction site safety management is operated on an independent server platform in a remote location. The server platform receives the CCTV video data from the construction site based on the Open Network Video Interface Forum (ONVIF) protocol. In addition, the video data are input into the object recognition model of the construction site safety management system. ONVIF is one of the worldwide open industry forums, and is an open standard for the interface of physical IP-based products. In other words, ONVIF is used to standardize the communication between network-based video devices and enhance their interoperability. Hence, we developed the ONVIF standard interface module to receive construction site image data efficiently, as shown in Figure 10 [40].

**Figure 11:**
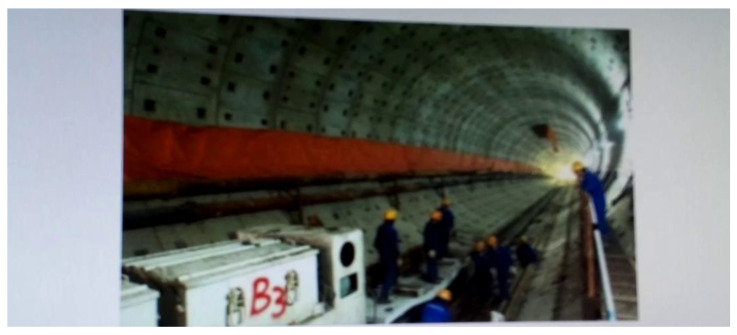
Video data reception test based on the ONVIF standard protocol.

Figure 11 shows the test structure for receiving the video data of a construction site based on the ONVIF standard protocol. The ONVIF server module for collecting the test videos was implemented using Raspberry Pi. The video data are received by the construction site safety management server platform, which operates the object recognition model, through the ONVIF client module.

### 4.2. Evaluation Indices

In this study, the precision, recall, and F1 score were used as indices to verify the performance of the created models. Precision indicates the ratio of the actual “true” objects to the objects classified as true by the object recognition model. Equation (1) below shows its mathematical expression. In other words, precision signifies the proportion of the actual true objects among the objects classified by the model, and is also called positive predictive value (PPV).

(1) Precision=〖〖TP〗〗/〖〖TP+FP〗〗=〖〖TP〗〗/〖〖All detection〗〗

Recall is the proportion of objects the model predicted to be true among the actual true objects. Recall can be expressed as Equation (2) below, and indicates the proportion of the true objects among the actual values (all ground truths) that represent the corresponding object information in the actual construction site images.

(2) Recall=〖〖TP〗〗/〖〖TP+FN〗〗=〖〖TP〗〗/〖〖All Ground Truths〗〗

In general, precision and recall are inversely proportional to each other, and the F1 score can be defined as the harmonic mean of the precision and recall. The F1 score is an index that can evaluate the performance of the model more accurately when the labeled data are imbalanced.

(3) F1 Score=2∗〖〖Precision∗Recall〗〗/〖〖Precision+Recall〗〗

The intersection over union (IoU) is an evaluation index used to measure the accuracy of the object recognition. The IoU numerically represents the degree of overlap between the location of the labeled object and the location of the object predicted by the object recognition model. In other words, an IoU value close to 1 indicates that the object recognition model has accurately predicted the location of the object (bounding box).

**Figure 12:**
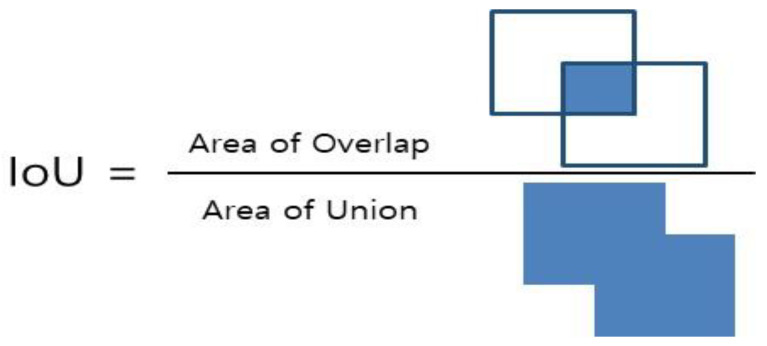
IoU (intersection over union) evaluation index for measuring the accuracy.

Figure 12 illustrates the concept of IoU in a diagram form. The area of union represents the entire area of the predicted bounding box and the ground-truth bounding box. The area of overlap represents the overlapped area between the predicted bounding box and the ground-truth bounding box. The IoU value is calculated by dividing the area of overlap by the area of union, and the performance of the model, such as the precision and recall, is measured based on this value. In general, the IoU threshold is set to 0.5, and Table 4 shows the performance measurement results of the model using a threshold value of 0.5.

The performance of the object recognition model was evaluated using workers and their safety helmets. That is, the performance was evaluated by the accuracy of the object recognition model in detecting the object (worker, helmet, etc.) in the validation image dataset. The total number of bounding boxes of the image dataset used to verify the object recognition model was 6535, which can be expressed as the sum of true positives (TPs) and false negatives (FNs). The number of false positives (FPs) varies depending on the performance of the object recognition model. Herein, TP refers to the case where the object recognition model accurately detects an object (e.g., when the object recognition model recognizes workers as workers in the image). FP implies that the object recognition model recognizes an incorrect object (e.g., when the object recognition model recognizes nonworker objects as workers in the image). FN implies that the object recognition model does not accurately recognize the object (e.g., when the object recognition model does not recognize the workers present in the image). “All detection” is the sum of TP and FP, and it implies all objects or the number of objects detected by the object recognition model (e.g., the number of objects classified as workers by the worker object recognition model). “All ground truths” can be expressed as the sum of TP and FN, which can be regarded as a labeled high-fidelity dataset used to train an object recognition model.

A precision–recall (PR) curve graph was utilized to assess the object recognition model performance developed in this study. We measured the data while decreasing the confidence level (the model threshold) of the object recognition model by 0.01 from 1 to 0.1; subsequently, we plotted a PR curve graph based on the measured data. The PR curve is a graph illustrated according to the precision of the recall values, in which the x-axis and y-axis represent the recall and precision, respectively. High performance in both precision and recall indicates an object recognition model with good performance. In general, the precision and recall are inversely proportional. Table 4 presents the PR curve for the construction site worker object recognition model, where the precision value does not rapidly decrease as the recall value increases, and both precision and recall values are numerically high. In the construction worker object recognition model, the precision value was 0.93 (93%), and the recall 0.73 (73%) when the threshold (confidence level) was 0.5.

Table 5 presents the PR curve of the PPE object recognition model. This graph also shows high numerical values for the precision, despite the increase in recall value. Specifically, the precision value was 0.89 (89%) and recall was 0.72 (72%) when the threshold (confidence level) value was 0.5, demonstrating excellent performance.

### 4.3. Simulation and Tests

In this paper, we have performed validation of our developed model. First, we conducted a test in a lab environment to check the object recognition model that determines whether workers are present at the construction site using the construction site integrated safety management system. The main objective of this model is to detect whether there are workers at the construction site. In the lab environment, we were able to verify that the model accurately detects the workers. In addition, the model was developed to count how many workers are in the camera image. Moreover, the second AI model detects the tilt of the head and torso and the bending of the knee joint. By incorporating factors such as the time and speed for the bending of each joint, the model was able to recognize the features more accurately. Finally, tests were performed to determine whether PPE was worn. Here, the model determined whether each worker was properly wearing a helmet. This model does not simply determine whether the helmet is recognized in the image, but also whether the worker is wearing the helmet properly.

**Figure 13:**
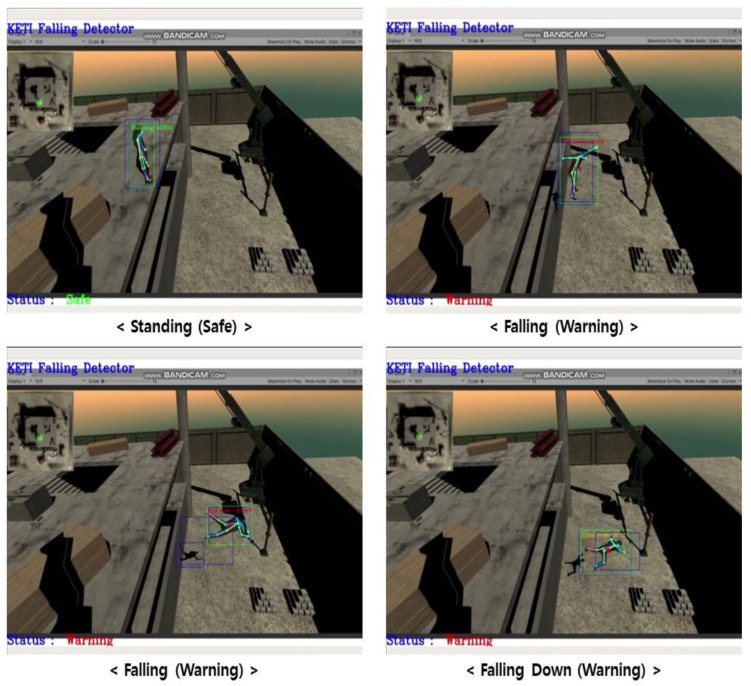
The verification of artificial intelligence object recognition model based on virtual environment (Fall detection of workers).

Furthermore, we performed verification of the artificial intelligence model that we developed based on the virtual environment. Figure 13 shows the results of testing a worker fall injury detection algorithm by simulating images of the fall of construction workers in a virtual environment. In the virtual environment, efficient detection of worker objects could be observed, and appropriate recognition of a worker fall detection model as a dangerous situation when the worker fell or tripped over can be observed in Figure 13. Verification of the performance of the object recognition model, which was developed based on the virtual environment, and structure and procedure of applying the optimized object recognition to the construction site are essential schemes proposed in this study. In particular, simulating and performing tests on worker falls and fall injuries, which are difficult to re-enact by actual humans, will be efficient algorithm verification methods.

**Figure 14:**
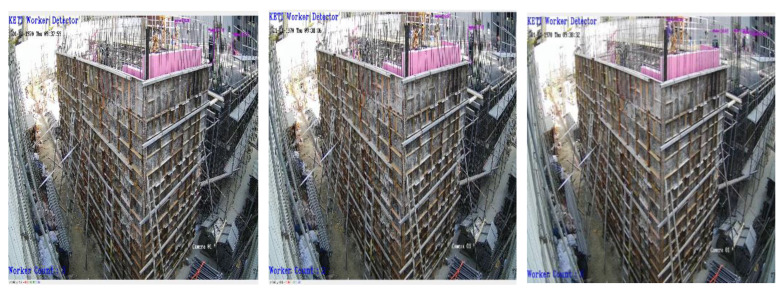
The construction site empirical results of worker object recognition AI model (Model 1).

**Figure 15:**
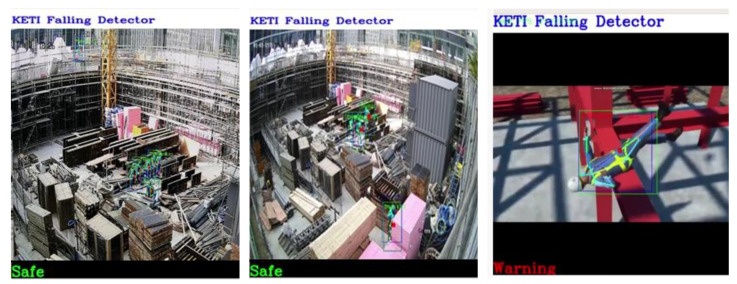
The construction site demonstration result of fall detection AI model of worker (Model 2).

**Figure 16:**
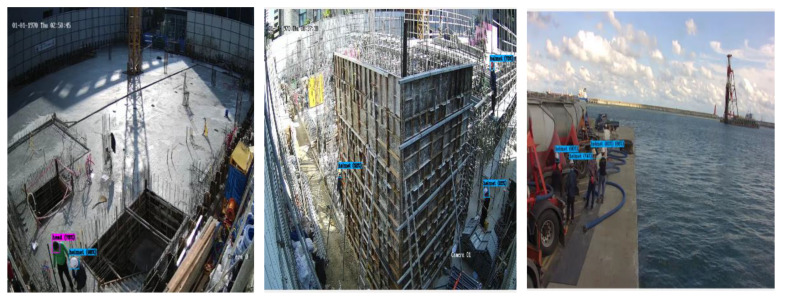
The construction site empirical results of PPE detection AI model (Model 3).

Figure 14, Figure 15 and Figure 16 present the results of field demonstrations of the developed algorithms. The initial object recognition model was a model trained with data only documented at the front of the camera, which was unsuccessful in recognizing images received from a camera installed laterally at a construction site. This study enhanced the rate of object recognition by additionally collecting lateral image data from virtual environments and actual construction sites and training with them (data-centric AI).

Figure 14 shows the results of testing the object recognition model that detects workers from construction sites, which efficiently recognized far-flung worker objects in empirical environments. Figure 15 shows the demonstration results of the worker fall detection model. Although an actual worker’s fall could not be detected in an empirical environment, efficient detection of the worker’s skeletons was observed. In addition, the walking or sitting states of workers were effectively detected. Finally, Figure 16 presents the empirical test results of the PPE detection model. Despite the images being in bird’s-eye view, it effectively determined workers with safety hats and no safety hats.

## 5. Conclusions and Future Research Direction

In this study, we conducted research on the development of an integrated safety management system for preventing safety accidents at construction sites by creating image recognition models using synthetic data based on transfer learning. In particular, an object recognition model was created to detect workers wandering around the construction site outside working hours or people trespassing at the site. In addition, a model was created to recognize dangerous situations in advance, such as the fall (falling off a structure or falling down) of a worker, by sensing the fall (falling down) of workers who are working at a height. The model that detects worker falls (falling off a structure or falling down) can be utilized to respond to a worker accident within the shortest amount of time possible after it occurs. Finally, an object recognition model was developed to determine whether construction workers are properly wearing personal protective equipment such as helmets. This object recognition model determines whether workers are complying with the rules for wearing safety helmets. Based on the developed object recognition models, an integrated safety management application was developed to manage the safety of workers at the construction site. In this paper, the collection of construction site image data, the preprocessing of the collected data, the image data augmentation method, the structure of the datasets, the virtual environment-based data collection and simulation environment, the structure of the training data for each model, the procedure for the development of the object recognition models, the implementation of the safety management system, and test results are comprehensively described. The developed and tested object recognition models will be applied to actual construction sites, and it is expected that these models will significantly reduce the number of accidents involving construction workers. As a direction of future research, we will conduct a study to optimize each model and maximize the performance based on the actual construction site. Furthermore, we will conduct more in-depth research on collecting and augmenting image data based on a virtual environment.

## Figures and Tables

**Table 1:** Development environment.

Hardware Server Platform		Software Server Platform	
CPU	AMD Ryzen 9 3950x	Operating System	Ubuntu 18.04
RAM	64 GB	Kernel	5.4.0-54-generic
GPU	GeForce RTX3080	Language	Python 3.8.0
LAN	Gigabit Ethernet	Virtual Environment	Anaconda 4.9.2
Main Storage	NVMe 1 Tb	Vision Library	OpenCV 4.2.0
Data Storage	HDD 4 TB	TensorFlow	1.15.2

**Table 2:** Image binarization.

Before Binarization	After Binarization
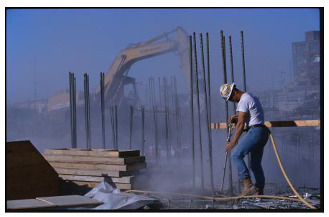	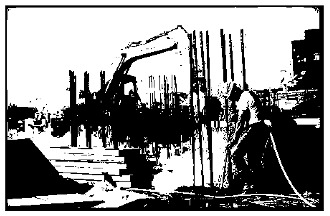

**Table 3:** Image segmentation.

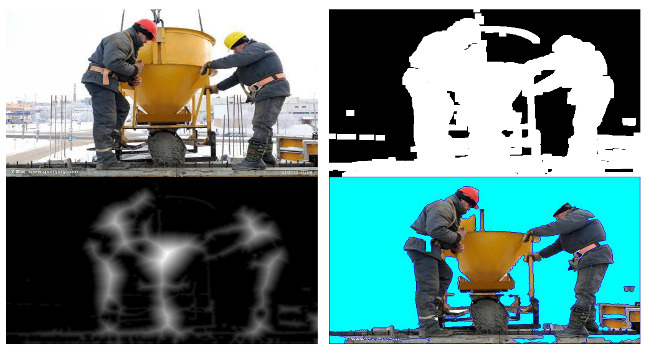

**Table 4:** PR curve graph of worker object recognition model (worker detection).

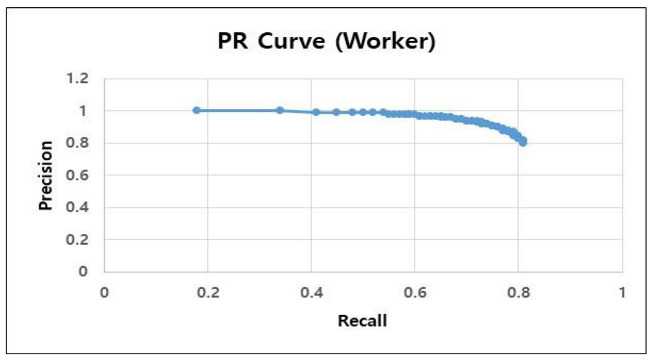

**Table 5:** PR curve graph of PPE object recognition model (PPE detection).

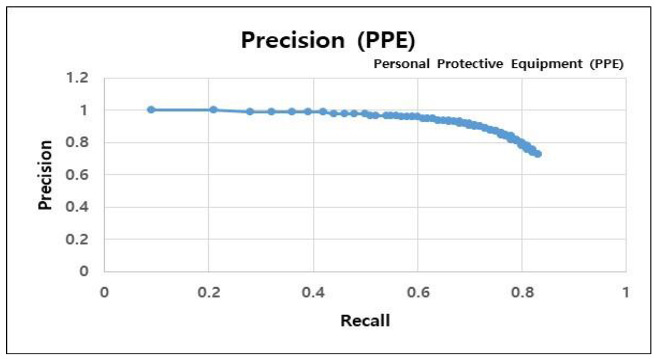

## Data Availability

Not applicable.
